# The role of mitochondrial energy metabolism in drug resistance and prognosis of lung adenocarcinoma: a multi-omics and machine learning strategy for predictive and personalized therapy

**DOI:** 10.3389/fmed.2026.1892773

**Published:** 2026-07-31

**Authors:** Chengyang Wu, Rui Jiao, Hanyu Yan, Yichen Sun, Tao Zhang, Yimeng Zhang, Xiaolong Yan, Zhaoyang Wang

**Affiliations:** 1 Department of Thoracic Surgery, Tangdu Hospital, The Fourth Military Medical University, Xi’an, China; 2 School of Medicine, Northwest University, Xi’an, China; 3 Department of Ophthalmology, Tangdu Hospital, The Fourth Military Medical University, Xi’an, China

**Keywords:** drug resistance, immunotherapy, lung adenocarcinoma, machine learning, mitochondrial energy metabolism

## Abstract

**Background:**

As the leading histological form of lung cancer, lung adenocarcinoma (LUAD) displays considerable intratumoral heterogeneity, frequent therapeutic resistance, and an unfavorable clinical outcome. Although rewiring of mitochondrial energy metabolism is known to drive tumor progression and treatment failure, a comprehensive understanding of its dual role in LUAD drug resistance and prognosis has yet to be established. Here, we built a robust predictive signature that integrates mitochondrial metabolism with drug resistance through multi-omics integration and machine learning frameworks.

**Methods:**

We explored single-cell RNA sequencing profiles together with TCGA-LUAD transcriptomic data. Weighted gene co-expression network analysis (WGCNA) was applied to extract gene modules linked to mitochondrial-related genes (MRGs) and drug resistance-related genes (DRGs). From these, a five-gene (KLF4, KLF10, CAT, ALDOA, HLA-DRA) prognostic classifier, designated MDrisk, was formulated using LASSO-Cox regression and 101 combinations of 10 machine learning algorithms.

**Results:**

The MDrisk model demonstrated reliable and precise prognostic capacity across training, internal test, and external GEO cohorts, serving as an independent risk factor. Elevated MDrisk scores correlated with an immunosuppressive microenvironment, higher tumor mutational burden, distinct copy-number alteration profiles, and decreased drug sensitivity in computational predictions. *In vitro* experiments further validated that silencing ALDOA—a central component of the signature—suppressed the proliferation, migration, and invasive capacity of LUAD cells.

**Conclusion:**

The MDrisk signature derived from mitochondrial energy metabolism and drug resistance may be useful for distinguishing prognosis, immune contexture, and computationally inferred drug susceptibility in LUAD. It may offer a tool for further exploration of individualized therapy and sheds light on the interplay between metabolic dysregulation and antitumor immunity.

## 1 Introduction

Lung adenocarcinoma (LUAD) remains the most prevalent and lethal subtype of lung cancer worldwide, characterized by high heterogeneity, frequent drug resistance, and unsatisfactory long-term prognosis (1, 2). Despite advances in chemotherapy, targeted therapy, and immunotherapy, many patients develop resistance and experience disease progression. This reality underscores the pressing demand for identifying novel biomarkers that can predict outcomes and pinpoint actionable therapeutic vulnerabilities.

Mitochondrial energy metabolism acts as a central regulator of tumor cell proliferation, survival, and therapeutic response (3). Abnormal mitochondrial function reshapes the tumor microenvironment, suppresses anti-tumor immunity, and modulates the expression of immune checkpoints, thereby reducing sensitivity to immunotherapy (4). Mounting evidence indicates that mitochondrial metabolic reprogramming is closely associated with drug resistance and poor prognosis in multiple malignancies (5, 6). Reprogramming of mitochondrial metabolism sustains rapid growth, promotes invasion, and drives resistance to multiple treatments in LUAD (7), yet the clinical relevance and underlying mechanisms linking mitochondrial metabolism to drug resistance and patient prognosis remain incompletely understood.

Multi-omics integration and machine learning have emerged as powerful strategies to dissect complex molecular networks and build robust predictive models for cancer classification and risk stratification. Single-cell transcriptomics, in particular, provides unprecedented resolution for characterizing cell-type-specific transcriptional programs and ligand–receptor crosstalk within the tumor microenvironment (TME). In the current investigation, we dissected single-cell RNA sequencing datasets to pinpoint gene modules closely associated with MRGs and DRGs in LUAD. Through overlapping multi-source gene sets and LASSO-Cox regression, we constructed a five-gene (KLF4, KLF10, CAT, ALDOA, HLA-DRA) prognostic signature, termed MDrisk, and validated its predictive performance using multiple machine learning algorithms across independent cohorts.

We further verified that the MDrisk classifier operates as an independent prognostic determinant, strongly interrelated with the extent of immune infiltration, expression of immunological checkpoints, tumor mutation burden (TMB), and copy number variations (CNVs). High MDrisk scores were linked to an immunosuppressive microenvironment and reduced sensitivity to multiple therapeutic agents as estimated by computational algorithms. Functional experiments confirmed that ALDOA, a key glycolytic enzyme in the signature, promoted LUAD cell proliferation, migration, and invasion.

Taken together, our work establishes a crucial nexus between mitochondrial energy metabolism, drug resistance, and immune modulation in LUAD. The MDrisk model may be a useful tool for prognostic estimation and could provide a basis for future research into individualized therapeutic decision-making, and furnishes novel perspectives on metabolic-immune interplay and interventional strategies in LUAD.

## 2 Materials and methods

### 2.1 Data collection and processing

Clinical and transcriptomic profiles associated with LUAD were retrieved from The Cancer Genome Atlas (TCGA) database. The TCGA-LUAD cohort comprised 535 tumor samples and 59 adjacent normal tissue samples with complete transcriptomic and clinical follow-up data. All patients with histologically confirmed LUAD and available survival information (overall survival ≥ 30 days) were included; samples without survival data or with incomplete clinical annotation were excluded. Key clinical variables encompassed age, gender, pathologic TNM stage, and overall survival status and duration. Single cell omics datasets were obtained from the Gene Expression Omnibus (GEO) database and integrated by matching sample identifiers, genomic annotations, and single cell transcriptome profiles. For external validation, the GSE31210 dataset (*n* = 226, platform GPL570) and the GSE72094 dataset (*n* = 442, platform GPL15048) were retrieved, with inclusion restricted to samples having complete survival follow-up records and gene expression profiles. Single cell RNA sequencing data were processed and analyzed using the R package “Seurat” (The R Foundation for Statistical Computing, Vienna, Austria). Dimensionality reduction and cell clustering were performed using the FindNeighbors and FindClusters functions implemented in Seurat, followed by visualization via uniform manifold approximation and projection (UMAP). Copy number variation (CNV) analysis was conducted using the R package “inferCNV,” with normal B cells applied as the reference population to evaluate CNV levels across distinct epithelial cell subsets. The predictive performance and robustness of the MDrisk model were further validated in the GSE31210 and GSE72094 datasets, as well as in training and internal testing cohorts (8–10).

### 2.2 Gene screening

To carry out candidate gene screening, inter-sample correlation assessment and clustered co-expressed gene module recognition were achieved through weighted gene co-expression network analysis (WGCNA). Network construction for TCGA LUAD transcriptome data was completed with the aid of the WGCNA package in R language. The scale-free network property was guaranteed by selecting the best soft threshold power value. Gene sets remarkably associated with MRGs and DRGs were further distinguished via AUC analysis. These candidate genes were then overlapped with differentially expressed genes (DEGs) and prognosis-associated genes in the TCGA dataset to obtain a final set of key overlapping genes for subsequent model construction.

### 2.3 Immunological and therapeutic performance analysis

To eliminate biases introduced by single-algorithm reliance, a multi-algorithm integrated analytical framework was adopted. Four computational algorithms were incorporated in this workflow: TIDE, CIBERSORT, XCell, and ESTIMATE. Among them, CIBERSORT was leveraged to quantify tumor-infiltrating immune cell proportions and distribution discrepancies between the high- and low-MDrisk cohorts. The R estimate package was implemented to quantify stromal and immune fractions as well as deduce tumor purity in the tumor microenvironment (TME). GSVA was further conducted to explore differential enrichment trends of predefined biological gene signatures in individual samples. Quantitative indices including immune score, stromal score and ESTIMATE score were subsequently computed to systematically depict the stromal and immune landscape of tumor specimens. TIDE scores were further applied to computationally infer predict the potential immunotherapeutic responses of different risk subgroups (11). Differential expression levels of immune checkpoint genes across distinct MDrisk subgroups were compared via the Wilcoxon rank-sum test, and statistical significance was defined as *P* < 0.05.

### 2.4 Functional enrichment analysis

Gene ontology (GO) functional annotation and KEGG pathway enrichment analyses were executed relying on four R packages, namely clusterProfiler, org.Hs.eg.db, enrichmentPlot, and ggplot2. This batch of enrichment analyses aimed to screen biological functions and signaling pathways markedly enriched in separate MDrisk subgroups, which helped dissect the intrinsic biological processes correlated with patient prognosis. Subsequently, GSEA was introduced to contrast pathway enrichment landscapes between the high- and low-MDrisk cohorts, uncovering 64 pathways with distinct enrichment tendencies. GSVA was next utilized to assess enrichment status at the individual sample level. Statistical significance was jointly defined by three criteria: *P* < 0.05, FDR-adjusted q-value < 0.05, and absolute log_2_ fold change exceeding 0.1.

### 2.5 MDrisk construction

Ten independent machine learning approaches along with their 101 combined algorithm schemes were constructed to build a robust, high-performance MDrisk prognostic signature. The candidate algorithms covered random survival forest (RSF), elastic net (Enet), LASSO, ridge regression, stepwise Cox regression, CoxBoost, plsRcox, supervised principal components (SuperPC), survival support vector machine (survival-SVM), and generalized boosted regression modeling (GBM). The entire modeling workflow contained three sequential steps: (I) Univariate Cox regression analysis screened prognosis-relevant genes within the TCGA-LUAD cohort; (II) 10-fold cross-validation was implemented to train and assess each of the 101 combined algorithm models; (III) External validation of all candidate models was conducted using two independent datasets, GSE31210 and GSE72094. Detailed parameter configurations and overfitting prevention measures are as follows: For LASSO and Elastic Net implemented via the glmnet package, we set the alpha grid as seq(0, 1, 0.1) and selected optimal λ through 10-fold cross-validation; ridge regression adopted a fixed alpha value of 1e-4. Bidirectional stepwise Cox regression utilized an entry P threshold of 0.05 and stay P threshold of 0.10. The random survival forest (RSF) was built with 1,000 trees, mtry set as the square root of total feature counts, and node size fixed at 15. CoxBoost contained 200 boosting iterations with a penalty factor of 0.01, while plsRcox determined latent component numbers via cross-validation. SuperPC retained the top 50 principal components, survival-SVM adopted a linear kernel with cost parameters optimized by cross-validation, and GBM was configured with 500 trees, an interaction depth of 3, and a shrinkage factor of 0.01. The 101 combined pipelines were constructed via a two-tier framework: univariate Cox regression served as the preliminary filtering step, followed by all single-algorithm and pairwise combined schemes of the 10 base models. Crucially, no hyperparameter adjustment was performed using internal test or external GEO datasets throughout the entire modeling process. To block information leakage across cohorts, we randomly split the original TCGA-LUAD dataset into a 70% training set and a 30% internal test set at the earliest analytical stage, while two external validation datasets (GSE31210, GSE72094) remained completely isolated and never participated in any feature screening, hyperparameter tuning, or model training. All WGCNA, univariate Cox and LASSO analyses were solely executed on the training cohort; test and external datasets were blinded until final performance evaluation. Clinical covariates including age, gender, and TNM stage were analyzed separately after risk score calculation and were never incorporated into gene feature screening workflows. For overfitting mitigation, L1 and L2 regularization embedded within LASSO and Elastic Net compressed redundant gene coefficients to simplify model complexity. All feature selection and hyperparameter searching relied on 10-fold cross-validation on the training set to select optimal parameters. Multi-cohort validation confirmed stable C-index and AUC metrics across independent patient populations, ruling out overfitting to a single dataset. We also progressively reduced feature dimensions from thousands of candidate genes down to a concise five-gene signature to lower model complexity, and we avoided repeated resampling or duplicate training on identical sample subsets during performance assessment.

### 2.6 Survival analysis and nomogram establishment

Sample grouping was implemented following the preset research scheme, including a training set, test set, and two external validation datasets GSE31210 and GSE72094. The optimal risk-score cutoff was determined using the surv_cutpoint function from the survminer R package, which exhaustively searches for the threshold yielding the most statistically significant separation in overall survival between the two groups based on the maximally selected rank statistics. The optimal threshold of the MDrisk score was applied to separate enrolled individuals into high- and low-risk populations. Inter-group survival disparities were examined via KM survival analysis relying on the survminer package in R. Subsequent univariate and multivariate Cox regression analyses validated the independent prognostic potency of the MDrisk signature alongside conventional clinical factors (age, gender, TNM stage). A combined nomogram integrating the risk score and critical clinical indicators was generated to refine prognostic prediction and quantify survival outcomes for LUAD cases. C-index and calibration curve analyses were further applied to quantify the nomogram’s predictive reliability (12).

### 2.7 Evaluation of therapeutic drug responsiveness

For auxiliary clinical decision-making regarding treatment regimens, drug response levels were computationally estimated for LUAD patients stratified by MDrisk score with the assistance of the oncoPredict R toolkit. IC50 metrics were derived for individual samples, followed by Wilcoxon test to inspect significant disparities in drug sensitivity between the two risk subgroups.

### 2.8 Genomic variation in MDrisk subgroups

The R package maftools was applied to dissect genomic mutation profiles across the two MDrisk subgroups. Oncogenic mutation waterfall diagrams were plotted to exhibit the most frequently mutated genes in LUAD patients assigned to the high-risk and low-risk cohorts, respectively. Tumor mutational burden (TMB) was quantified for each sample, with subsequent analyses implemented to probe its correlation with the MDrisk score as well as its independent prognostic utility. Copy number variation (CNV) profiling was further conducted to characterize genomic aberrations occurring in hub genes from the MDrisk signature and interpret their prognostic relevance.

### 2.9 Quantitative real-time polymerase chain reaction (qRT-PCR)

RNA extraction was performed using TRIzol reagent in accordance with the established methods (13). Reverse transcription of isolated RNA to cDNA was completed via the PrimeScript RT reagent kit. The SYBR Green master mix was adopted for qPCR reactions running on the Bio-RAD iQ5 quantitative PCR instrument from the United States. Detailed primer sequences used for this qPCR assay can be found in the Supplementary Table.

### 2.10 Western blot assay

The samples were processed according to the experimental protocol (14). The primary antibodies were ALDOA (1:1000, Proteintech, 67453-1-Ig), Catalase (1:1000, Abclonal, A11220), HLA-DRA (1:10000, Proteintech, 17221-1-AP), KLF10 (1:500, Abcam, ab73537), KLF4 (1:1000, Abcam, ab215036), and VINCULIN (1:500, Abcam, ab129002).

### 2.11 CCK-8 proliferation assay

Each well of 96-well plates received stable cell suspensions containing 3 × 10^3^ cells diluted in 100 μL complete DMEM medium. CCK-8 working solution (Beyotime, Cat No. C0038) was introduced separately at 0, 24, 48, and 72 h, and samples were incubated away from light at 37 °C over a 1.5-h period. A Bio-Rad microplate reader was applied to detect absorbance signals at the 450 nm wavelength. The assay was independently repeated three times biologically, while six parallel technical replicates were arranged within every group.

### 2.12 Wound-healing experiment

Six-well plates were seeded with stable cells and cultured to reach a confluency range of 90%–100%. A uniform linear scratch was introduced by scraping the cell monolayer with a 200 μL pipette tip. Following PBS (Solarbio) washing steps, cells were cultured in DMEM without fetal bovine serum. Wound closure images were acquired at 0, 24, and 48 h under an inverted microscope (Olympus, 100 × magnification). The wound healing rate was quantified accordingly. Three independent biological replicates were conducted.

### 2.13 EdU incorporation assay

Cell proliferative capacity was detected with the BeyoClick™ EdU-594 detection kit (Beyotime, Cat No. C0078S, Shanghai, China), and all experimental manipulations strictly complied with the official manufacturer’s instructions. Cells were plated into 24-well plates and exposed to 50 μM EdU working solution at 37 °C over a 2-h incubation period. Afterwards, cell fixation and permeabilization were performed sequentially, followed by Apollo staining and DAPI counterstaining. Fluorescent signals of EdU-positive cells were captured with a Leica laser scanning confocal microscope at 200 × magnification. The proliferation rate was quantified as the proportion of EdU-labeled cells relative to all DAPI-stained total cells. Three independent biological replicates were performed.

### 2.14 Transwell invasion assay

Matrigel diluted 1:8 (BD Biosciences) was used to pre-treat 24-well Transwell chambers with 8.0 μm pores (Corning Costar, catalog number 3422). Cell suspensions consisting of 1 × 105 stable cells in 200 μL serum-free DMEM were placed in the upper chamber, while the lower reservoir received 600 μL full DMEM medium with 10% FBS serving as chemotactic attractant. After 48 h incubation, non-invasive cells attached to the upper side of the polycarbonate membrane were cleared. Invaded cells were fixed with 4% paraformaldehyde, stained with 0.1% crystal violet (Solarbio), and photographed (Olympus, 100 × magnification). Three independent biological replicates were carried out, with three random fields counted per group.

### 2.15 TMRM staining for mitochondrial membrane potential (ΔΨm) visualization

A549 and H358 cells stably expressing shControl (Con) or shALDOA were seeded into 24-well plates at a density of 5 × 10^4^ cells per well and cultured overnight. Cells were incubated with 100 nM TMRM (Tetramethylrhodamine, Methyl Ester, Perchlorate; Invitrogen, Cat. No. T668) in serum-free DMEM at 37 °C for 30 min in the dark. After incubation, cells were gently washed twice with pre-warmed PBS and immediately imaged using a Leica laser scanning confocal microscope (Leica TCS SP8, Wetzlar, Germany) with TRITC filter sets at 200 × magnification.

### 2.16 MitoSOX staining for mitochondrial reactive oxygen species (ROS) visualization

Mitochondrial superoxide levels were visualized using the MitoSOX Red reagent (Invitrogen, Cat. No. M36008). A549-shCon, A549-shALDOA, H358-shCon, and H358-shALDOA cells were plated in 24-well plates and allowed to attach overnight. Cells were washed once with PBS and then loaded with 5 μM MitoSOX Red in serum-free DMEM for 10 min at 37 °C in the dark. After staining, cells were gently washed twice with pre-warmed PBS and directly imaged using a Leica laser scanning confocal microscope (Leica TCS SP8, Wetzlar, Germany) with a rhodamine filter set at 200 × magnification.

### 2.17 OXPHOS-dependent ATP measurement

To evaluate the impact of ALDOA knockdown on mitochondrial oxidative phosphorylation, total cellular ATP levels were quantified using the Glycolysis/OXPHOS Assay Kit (Dojindo, G270) following the manufacturer’s instructions. Briefly, A549-Con, A549-shALDOA, H358-Con, and H358-shALDOA cells were seeded in 96-well white-walled plates at 1 × 10^4^ cells per well and cultured for 48 h. The culture medium was replaced with fresh medium containing the provided OXPHOS-specific substrate, and cells were incubated for an additional 2 h. Subsequently, cells were lysed, and the luciferase reaction mixture was added. Luminescence was recorded immediately using a microplate reader (BioTek Synergy H1). ATP concentrations were calculated from a standard curve and normalized to the protein content of each well, determined by BCA assay. Three independent biological replicates were performed, each with three technical replicates.

### 2.18 Immunohistochemistry (IHC)

Tumor tissues and matched adjacent non-tumor specimens were collected from 10 LUAD patients receiving diagnosis and surgical intervention at the Department of Thoracic Surgery, Tangdu Hospital, Fourth Military Medical University, Xi’an, China. Detailed clinical information of enrolled subjects is provided in Supplementary File 2. Formalin-fixed paraffin-embedded (FFPE) LUPE tissue blocks were sliced into consecutive 4-μm-thick sections. Following 2 h heating incubation at 80 °C, the slices underwent xylene dewaxing, gradient ethanol rehydration (100%, 95%, 90%, 85%, 75%), and ultrapure water washing sequentially. Antigen retrieval was conducted in Tris-EDTA buffer (Cat. No. P0084, Beyotime, Shanghai, China). Endogenous peroxidase activity was inhibited with peroxidase blocking reagent (P0100A, Beyotime). Subsequently, tissue sections were incubated with ALDOA primary antibody (dilution ratio 1:100, catalog 67453-1-Ig, Proteintech, Rosemont, IL, USA) overnight at 4 °C (15). Subsequent immunolabeling signals were visualized with the IHC detection kit applicable to rabbit and mouse primary antibodies (Cat No. PK10006, Proteintech, Rosemont, IL, USA). An Olympus VS200 digital whole-slide imaging scanner (Olympus, Tokyo, Japan) was utilized to acquire tissue micrographs. All research procedures strictly complied with the ethical guidelines outlined in the Declaration of Helsinki, and the study protocol obtained formal approval from the Ethics Committee of Tangdu Hospital, Fourth Military Medical University (approval No. TDLL-2025-07-07).

### 2.19 Statistical analysis

Statistical computations in this research were implemented with R software v4.4.1. Depending on data distribution and experimental design, multiple statistical approaches were selectively adopted, including Pearson correlation, Spearman correlation, Wilcoxon test, Kruskal-Wallis test, χ^2^ test and Student’s *t*-test. Statistical significance was defined at a threshold of *P* < 0.05.

## 3 Results

### 3.1 Single-cell transcriptomic profiling identified potential gene modules linked to MRGs and DRGs

**FIGURE 1:**
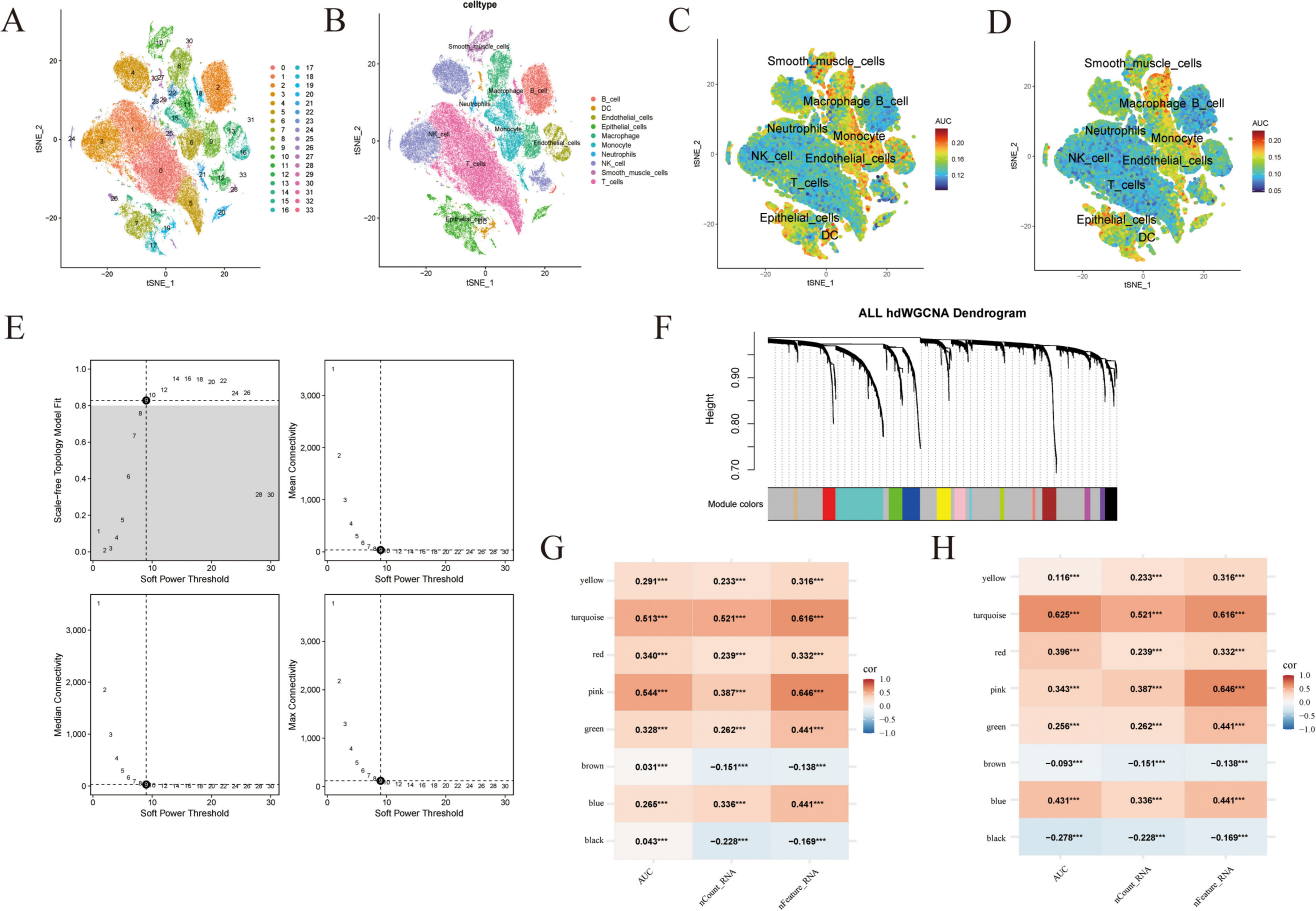
Screening of mitochondrial energy metabolism (MRGs) and drug resistance (DRGs) modules. **(A,B)** The t-SNE plot illustrates the various cell types that have been recognized using specific marker genes. **(C,D)** The t-SNE plot shows the AUC value of the MRGs and DRGs in each cell cluster. **(E)** Construction of the gene coexpression network based on the optimal soft threshold (β = 9). **(F)** Screening and identification of covariant gene modules. **(G,H)** Validation of gene modules closely correlated with mitochondrial energy metabolism and drug resistance phenotypes.***p < 0.001.

To delineate the gene expression pattern of LUAD, we analyzed a single-cell transcriptomic dataset derived from resected samples of LUAD patients (16). A total of 10 phenotypically distinct cell clusters were identified and annotated, including adaptive immune cells (T cells, B cells), innate immune cells [NK cells, monocytes, macrophages, dendritic cells (DCs), neutrophils], parenchymal cells (epithelial cells), and stromal cells (endothelial cells, smooth muscle cells) (Figures 1A, B). All cell subsets were well-separated, indicative of distinct transcriptomic signatures and reliable clustering results. AUC analysis validated the robustness of cell type annotation, with AUC values ranging from 0.05 to 0.20 across all cell populations (Figures 1C, D).

To construct a gene co-expression network, we performed hdWGCNA analysis. A soft threshold power was set at β = 9, consistent with the recommended criteria to satisfy the scale free topology criterion of the network (Figure 1E). Eight gene modules labeled with distinct colors (black, blue, green, brown, pink, red, turquoise, yellow) were identified, with module preservation scores ranging from 0.70 to 0.90, suggesting high stability of the co-expression network (Figure 1F). Further assessment was carried out on three indicators of the modules, namely AUC, nCount RNA and nFeature RNA. Candidate genes were filtered from the turquoise and pink modules according to the dual criteria *P* < 0.001 and *r* > 0.5; these gene sets possessed the tightest association with DRGs (Figure 1G). Meanwhile, genes within the turquoise module were identified as MRGs-associated genes (Figure 1H).

### 3.2 Construction and verification of the MDrisk model

**FIGURE 2:**
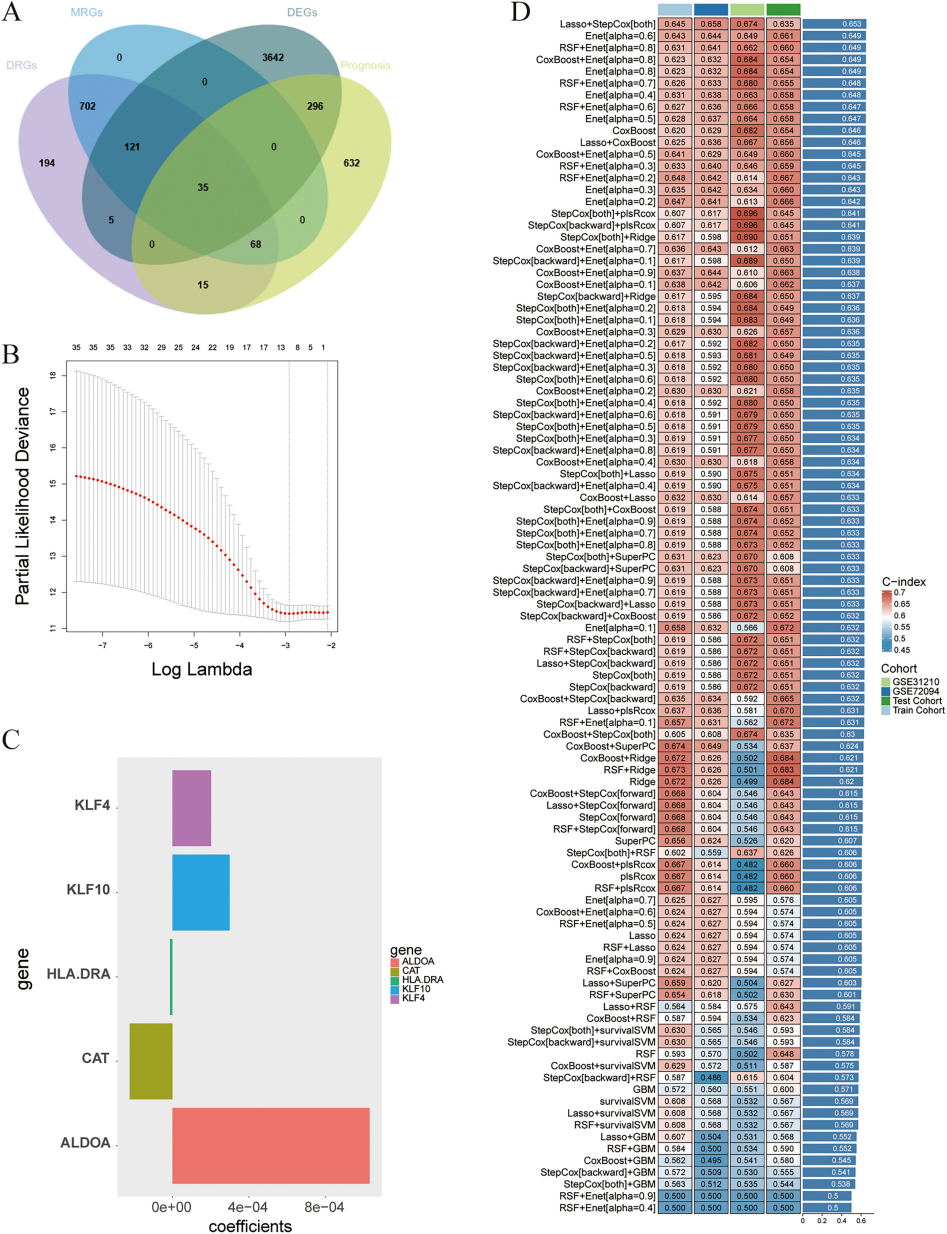
Construction of the MDrisk. **(A)** Genes associated with both mitochondrial energy metabolism (MRGs) and drug resistance (DRGs), along with the differentially expressed genes (DEGs) in normal tissues and lung adenocarcinoma (LUAD). **(B)** LASSO analysis for optimizing the selection of features and managing the complexity of models. **(C)** The final coefficients of the MDrisk following Stepwise Cox regression. **(D)** C-index value for all validation and training sets, which indicates the accuracy of the model’s predictions.

Through the intersection of 1140 DRG-associated genes and 926 MRG-associated genes separately identified by WGCNA, together with 1,046 genes linked to lung cancer prognosis and 4,099 differentially expressed genes between normal and LUAD tissues in the TCGA dataset, a total of 35 genes associated with LUAD prognosis, DRGs, and MRGs were identified (Figure 2A). By using LASSO-Cox regression analysis to further reduce overfitting, the five genes, including KLF4, KLF10, CAT, ALDOA, and HLA DRA, were identified to construct the optimal MRGs and DRGs related prognostic signature (Figures 2B, C). Based on the five candidate genes, 101 predictive models were generated via combinations of 10 distinct machine learning algorithms. The C-index values corresponding to each algorithm scheme were computed across multiple cohorts, covering the training set, internal test set, GSE31210 and GSE72094 external datasets. Among all combinations, the integrated Lasso and stepwise Cox regression model achieved the maximum average C-index of 0.653 and maintained stable predictive performance in every validation cohort. This optimal combination was finalized as the MDrisk model (Figure 2D).

**FIGURE 3:**
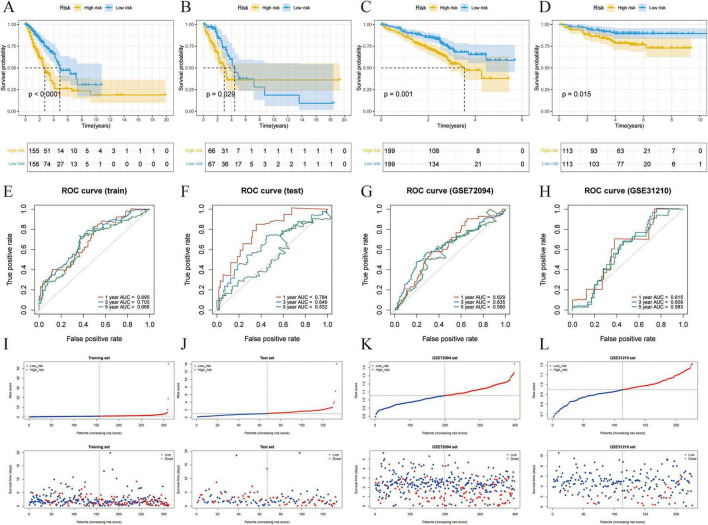
Verification of the MDrisk model. **(A–D)** Kaplan-Meier survival curves of the testing, training, GSE72094, and GSE31210 groups, showing the differences in survival based on MDrisk classification. **(E–H)** ROC curves for prediction of 1-, 3-, and 5-year survival. The curves demonstrate the prognostic value of the MDrisk. **(I–L)** Scatter plots illustrating the variation in survival time and risk scores among the groups.

**FIGURE 4:**
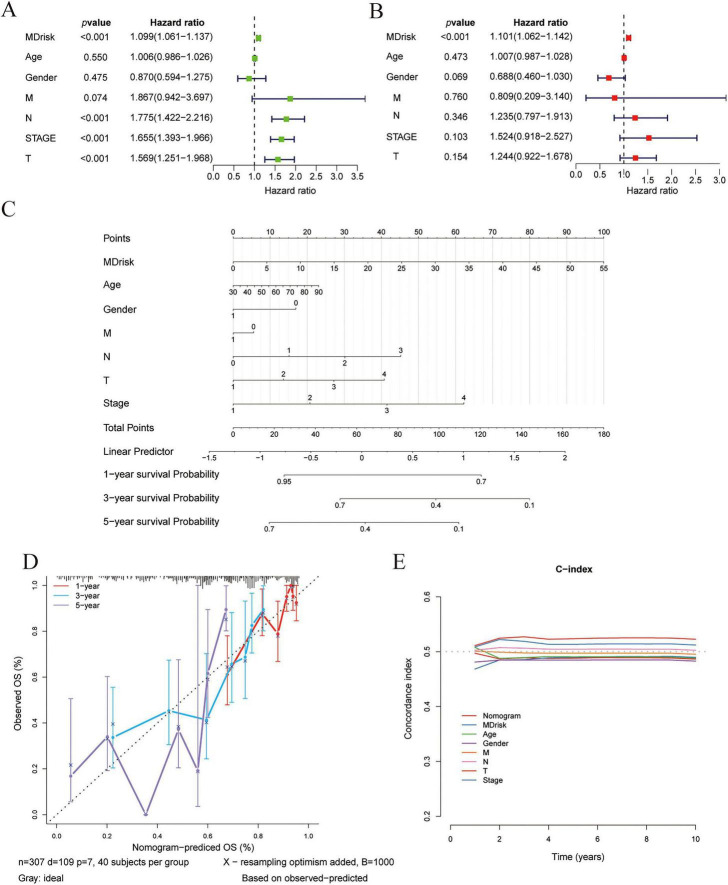
Development of the survival model nomograms. **(A,B)** Univariate **(A)** and multivariate **(B)** Cox regression analyses were used for statistical analysis of the nomogram, which included the MDrisk score and clinical aspects. **(C)** The nomogram combines several prognostic elements, resulting in a unique estimate of likelihood of survival. **(D)** A calibration graph was employed to assess the consistency of the forecasted and actual results, which ensures that the accuracy of the model is guaranteed. **(E)** A C-index value was calculated to test the predictive power of the nomogram.

Kaplan–Meier survival curves were plotted to assess the predictive efficacy of the MDrisk signature across all enrolled cohorts (Figures 3A–D). Significantly superior survival outcomes were observed in low-risk patients relative to their high-risk counterparts within each of the four cohorts. ROC curve analysis revealed that the MDrisk model yielded AUC values of 0.695, 0.703, and 0.666 for 1-, 3- and 5-year overall survival in the training cohort, reflecting reliable predictive capacity (Figure 3E). Satisfactory predictive power was also achieved in the three external validation cohorts for 1- and 2-year survival endpoints, with all calculated AUC values exceeding 0.6 (Figures 3F–H). Subsequent distribution analysis of survival duration and risk scores between the two subgroups verified that elevated MDrisk scores corresponded to markedly increased mortality risks (Figures 3I–L). Taken together, these outcomes confirm that the MDrisk signature exhibits robust prognostic prediction capability for LUAD patients. Age, sex, and TNM stage constitute well-established clinical prognostic markers for LUAD. Univariate and multivariate Cox proportional hazards regression models were adopted to clarify the independent prognostic value of the five-gene signature after adjusting for these clinical variables. Univariate Cox regression identified MDrisk score, T stage, N stage, and M stage as significant prognostic risk factors for LUAD (Figure 4A). Further multivariate analysis validated that only the MDrisk score served as an independent prognostic determinant (Figure 4B). These results highlight the potential advantages of the MDrisk signature for LUAD prognostic stratification. For further assessment of its clinical translation potential, a quantitative nomogram integrating the MDrisk score, age, sex, T stage, N stage, M stage and overall TNM stage was established (Figure 4C). Calibration curves exhibited strong consistency between nomogram-predicted survival probabilities and actual clinical observations (Figure 4D). Meanwhile, C-index calculations verified favorable predictive performance for both the standalone MDrisk model and the integrated nomogram (Figure 4E). In summary, the MDrisk signature is validated to be stable and accurate for prognostic assessment in LUAD patients.

### 3.3 Functional enrichment highlights immune activation and inflammatory signaling in the high-risk group

**FIGURE 5:**
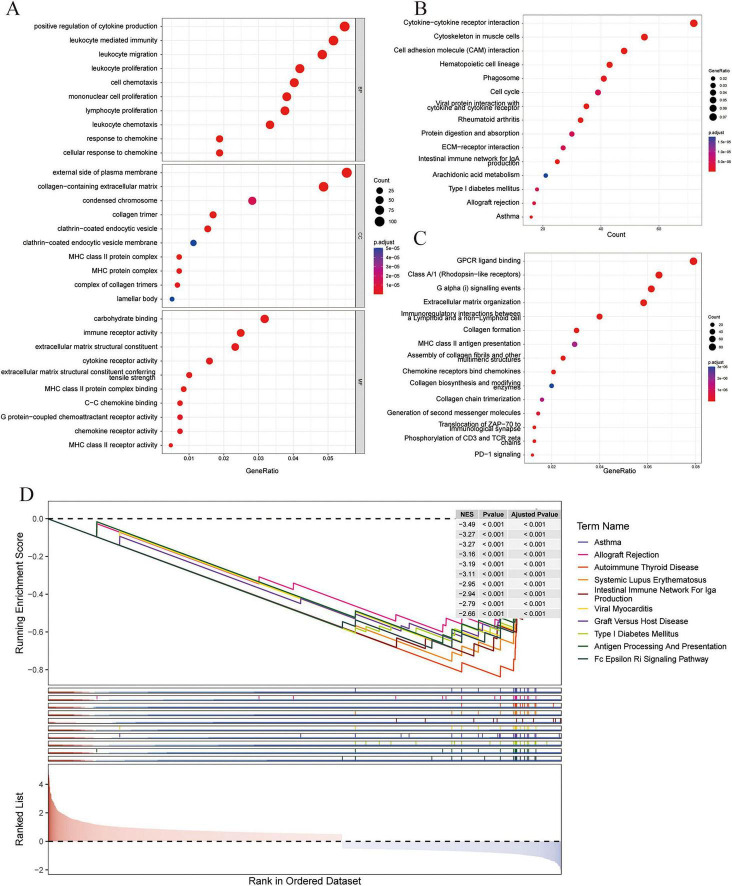
Functional enrichment of the MDrisk. **(A)** GO enrichment analysis of DEGs between the high-risk and low-risk MDrisk groups. **(B–D)** KEGG, Reactome, and GSEA identified the key biochemical processes and pathways that differentiate the high-risk and low-risk MDrisk groups.

Differentially expressed genes screened between high- and low-MDrisk subgroups underwent four enrichment strategies: GO, KEGG, Reactome, and GSEA. GO functional annotation displayed prominent enrichment entries covering multiple dimensions. In the biological process category, terms relevant to cytokine generation, leukocyte proliferation and chemokine-dependent immune responses were enriched; enriched cellular components encompassed plasma membrane and MHC protein complexes; enriched molecular functions contained immune receptor activity and chemokine receptor binding (Figure 5A). KEGG pathway enrichment pinpointed immune-associated cascades dominated by cytokine–cytokine receptor interaction and cell adhesion molecules (Figure 5B). Reactome enrichment analysis further validated such immune activation patterns, with MHC class II antigen presentation, chemokine receptor binding and PD-1 signaling standing out as significantly enriched items (Figure 5C). In line with the above enrichment trends, GSEA indicated a general suppression of diverse immune-related gene sets within the high-MDrisk cohort, including antigen processing and presentation as well as type I diabetes mellitus-linked immune pathways (Figure 5D). Overall, the multi-dimensional enrichment outcomes imply that divergent prognosis and treatment responsiveness across the two risk subgroups are partially driven by disrupted immune biological programs and signaling cascades.

### 3.4 The MDrisk score is associated with immune suppression and computationally inferred therapeutic response in LUAD

**FIGURE 6:**
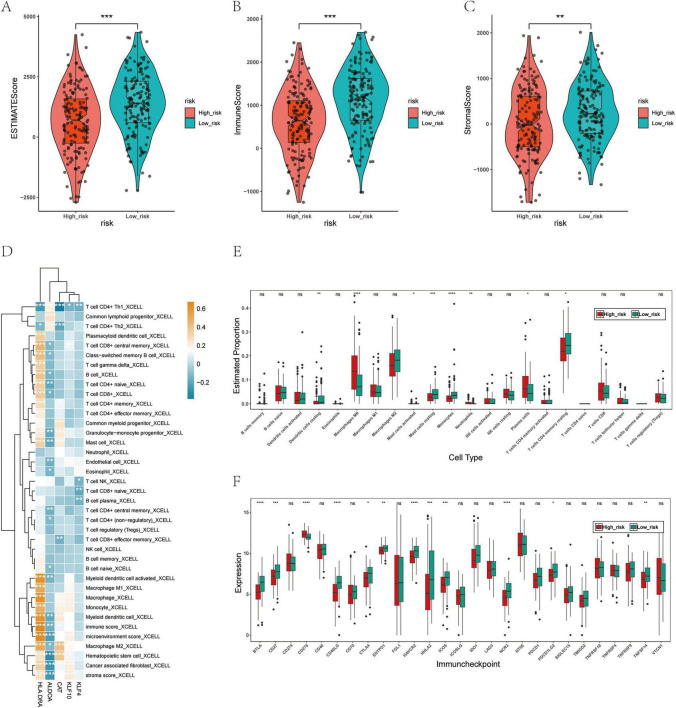
The immune characteristics of the MDrisk signatures. **(A–C)** Plots of the ESTIMATE, Immune, and stromal scores for quantifying tumor microenvironment complexity. **(D)** XCELL showed the connections between the MDrisk genes and immune cell types. **(E)** CIBERSORT was used to quantify the specific immune cell levels in each group. **(F)** Comparison of the immune checkpoint expression levels between the high-risk and low-risk MDrisk groups. *p < 0.05; **p < 0.01; ***p < 0.001; ****p < 0.0001.

**FIGURE 7:**
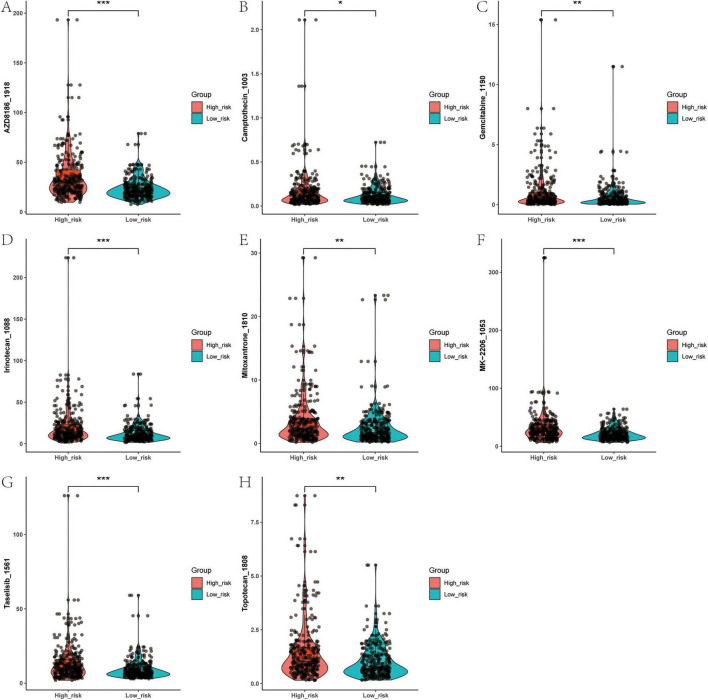
Evaluation of the performance of the MDrisk in predicting the effect of drug therapy. The half-maximal inhibitory concentration (IC50) values of eight anti-tumor drugs were compared between the high-risk (red) and low-risk (cyan) groups. **(A)** AZD8186_1918, **(B)** Camphotothecin_1003, **(C)** Gemcitabine_1180, **(D)** Irinotecan_1088, **(E)** Mitoxantrone_1810, **(F)** MK-2206_1053, **(G)** Taselisib_1561, and **(H)** Topotecan_1808. The high-risk subgroup exhibited significantly higher IC50 values for all tested agents, indicating reduced drug sensitivity compared with the low-risk subgroup. *p < 0.05; **p < 0.01; ***p < 0.001.

The correlation between the MDrisk score and immune activation as well as inflammatory signaling has been validated. On this basis, TIDE score, immune score, stromal score and ESTIMATE score were quantified systematically in high- and low-MDrisk subgroups to further dissect the computationally inferred prognostic capacity of the MDrisk signature in LUAD. Quantitative comparisons uncovered markedly elevated ESTIMATE scores in the high-risk cohort, which hints at inferior ICI treatment responsiveness as predicted by computational algorithms and unfavorable clinical outcomes for these patients (Figure 6A). By contrast, the low-risk subgroup obtained significantly higher immune and stromal scores, indicative of more abundant immune cell infiltration and lower tumor purity within this population (Figures 6B, C). These results support the capability of the MDrisk signature to computationally infer predict immune infiltration levels and potential immunotherapy responsiveness efficacy among LUAD patients. Next, correlation analyses were performed to explore the links between the five core prognostic genes (KLF4, KLF10, CAT, ALDOA, HLA-DRA) and the infiltration abundance of diverse immune cells (Figure 6D). Statistically meaningful associations were detected between these five genes and a total of 39 immune cell subtypes. To elaborate, HLA-DRA correlated significantly with macrophages, monocytes, myeloid dendritic cells and another 17 immune cell phenotypes; ALDOA showed correlations with cancer-associated fibroblasts, stromal score plus 14 additional immune cell types; CAT was associated with M2 macrophages, granulocyte-monocyte progenitors and 12 other immune subsets; KLF10 exhibited significant relationships with macrophages, neutrophils and 11 further immune cell populations; KLF4 was correlated with naive CD8+ T cells, plasma B cells and three other immune cell types. Immune landscapes were then contrasted across the two risk subgroups, revealing remarkable disparities in the fractions of numerous immune cell subsets (including M0 macrophages, activated dendritic cells and resting memory CD4+ T cells) and the expression levels of pivotal immune checkpoint genes. The high-risk group possessed larger proportions of proinflammatory and immunosuppressive cell populations. Meanwhile, multiple immune checkpoint genes (BTLA, CD27, CD40, CD40LG, NT5E) were distinctly upregulated in high-risk samples (Figures 6E, F). Collectively, these findings demonstrate that the high and low risk groups exhibit divergent immune landscapes, with the high-risk subgroup characterized by a more immunosuppressive tumor microenvironment. This immunological profile may correlate with the observed prognostic disparities and computationally inferred variable responses to immunotherapy between the two groups. Notably, these analyses rely entirely on *in silico* algorithms and do not constitute direct evidence of clinical immunotherapy benefit. In addition, drug sensitivity analysis revealed that eight therapeutic agents showed significantly higher predicted IC50 values in patients of the high-risk cohort (Figures 7A–H).

### 3.5 Somatic mutation and copy-number variation profiles between MDrisk groups

**FIGURE 8:**
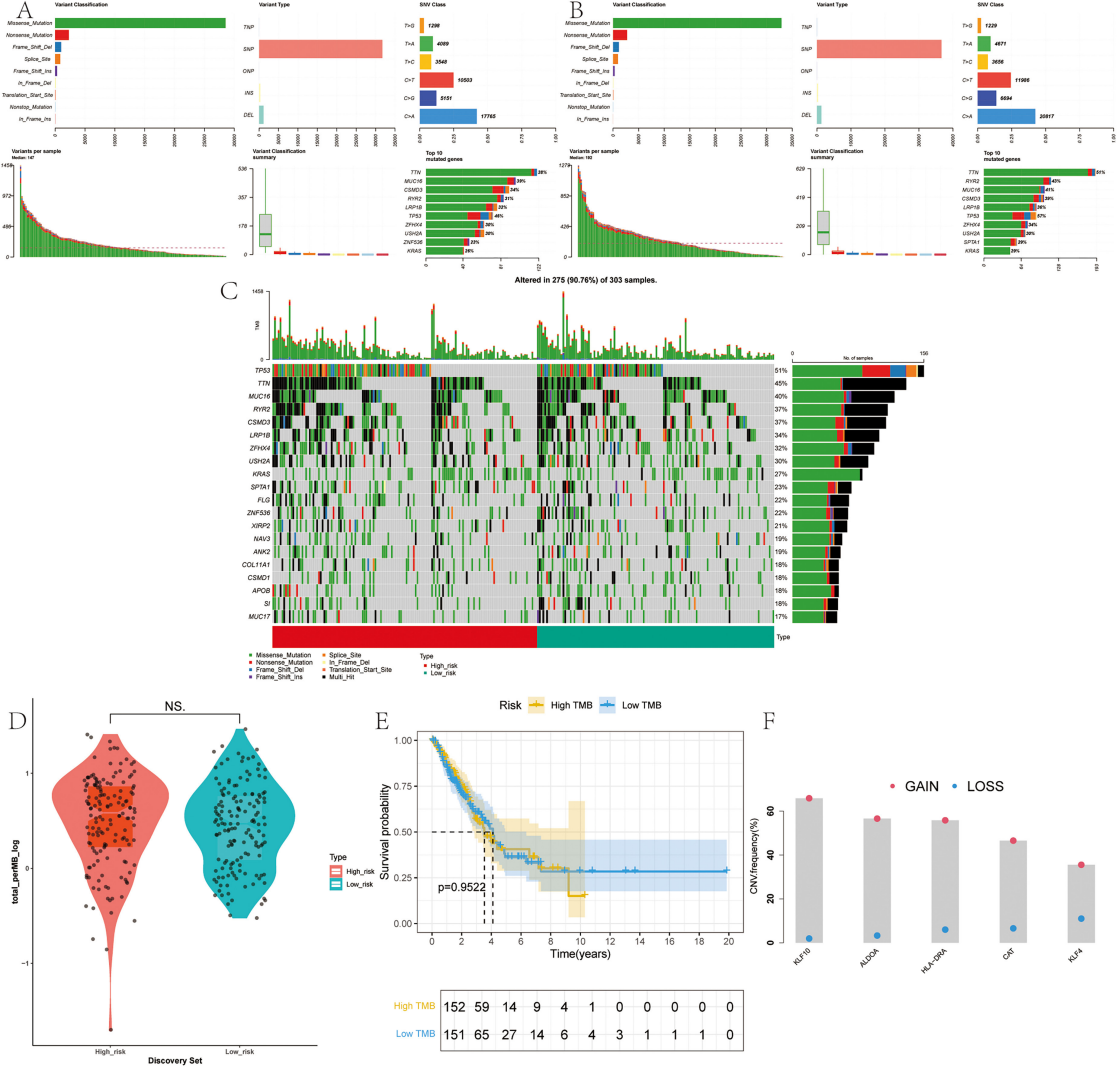
The predictive power of MDrisk for immunization status. **(A)** Landscape of somatic mutations in the high-risk subgroup. **(B)** Somatic mutation profiles of patients in the low-risk subgroup. **(C)** Classification and distribution of variant types for highly mutated genes in 30 lung adenocarcinoma (LUAD) cases. **(D)** Quantitative comparison of tumor mutation burden (TMB0 between high- and low-risk cohorts. **(E)** Prognostic significance of TMB stratification in LUAD patients (*P* = 0.65). **(F)** Frequencies of copy number variation (CNV) alterations for the five core genes in the MDrisk signature.

Mutational profiling revealed that missense mutations constituted the predominant mutation type within the high-risk cohort, and SNVs accounted for the largest proportion of all variant categories. Following the inspection of sample-specific mutation features and global variant-type distribution statistics, the top 10 recurrently mutated genes in the high-risk subgroup were screened out, among which TTN ranked prominently (Figure 8A). The corresponding mutation landscape of the low-risk group was further delineated (Figure 8B). Although the two subgroups exhibited identical variant classification systems and comparable SNV composition patterns, they diverged remarkably in sample-level mutation spectra, overall mutational signatures and the roster of the top 10 most frequently mutated driver genes.

For intergroup comparison of mutation spectra and evaluation of their prognostic influence on LUAD, waterfall charts were plotted to display the mutation landscape of highly mutated genes across risk subgroups (Figure 8C). TMB quantification uncovered a marked elevation in the high-risk population, which supports the capacity of the MDrisk model to predict TMB levels efficiently (Figure 8D). Even so, TMB-based patient stratification showed no statistically meaningful prognostic discrimination in this dataset (Figure 8E). Further CNV profiling was conducted on the core genes contained in the MDrisk signature. Copy number amplification dominated the genomic alterations of all five signature genes, and KLF10 presented the maximum amplification frequency (Figure 8F).

### 3.6 ALDOA knockdown inhibits proliferation, migration, invasion, and impairs mitochondrial function in LUAD

**FIGURE 9:**
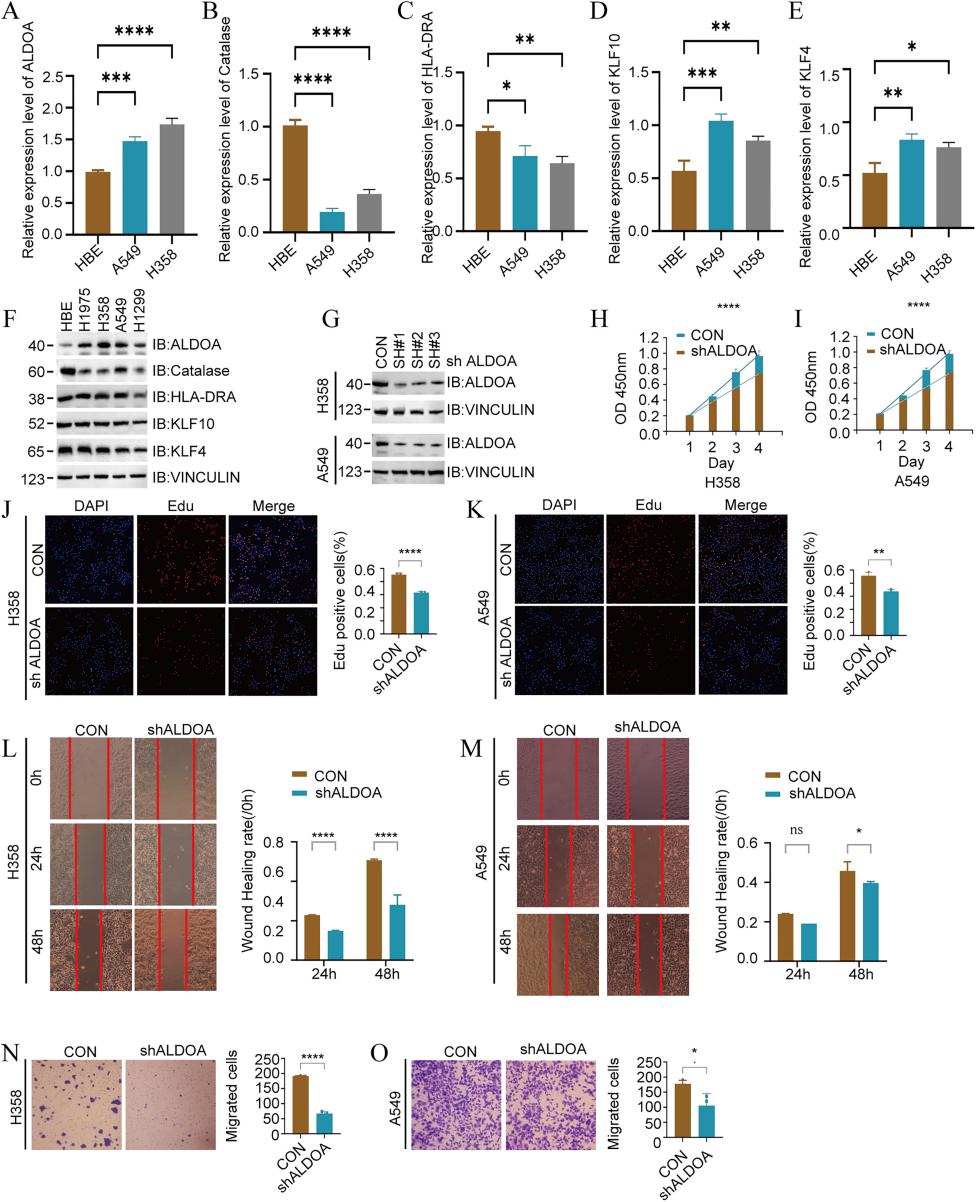
ALDOA drives progression of lung adenocarcinoma (LUAD) cells. **(A–E)** Relative mRNA expression levels of the five MDrisk signature genes in normal HBE cells and LUAD cell lines (A549 and H358). **(F)** Western blot detection of ALDOA, HLA-DRA, KLF4, KLF10, and CAT protein expression in HBE, A549, and H358 cells. **(G)** Western blot validation of efficient ALDOA knockdown in A549 and H358 cells. **(H,I)** CCK-8 assays revealed that ALDOA silencing markedly represses the proliferative ability of LUAD cells. **(J,K)** EdU staining further confirmed the inhibitory effect of ALDOA knockdown on cell proliferation. **(L,M)** Wound-healing assays demonstrated that ALDOA depletion impairs the migratory capacity of A549 and H358 cells. **(N,O)** Transwell invasion assays indicated that genetic silencing of ALDOA significantly suppresses the invasive potential of LUAD cells. *p < 0.05; **p < 0.01; ***p < 0.001; ****p < 0.0001.

We first examined the mRNA expression levels of five key MDrisk-associated genes in normal HBE cells and LUAD cell lines A549 and H358 (Figures 9A–E). The results showed that ALDOA, KLF10, and KLF4 were significantly upregulated at the mRNA level in LUAD cells, whereas HLA-DRA and CAT were downregulated compared with normal control cells. Western blot analysis confirmed that ALDOA protein was markedly increased in LUAD cells, consistent with its role as a key risk gene in our MDrisk model (Figure 9F).

Given that ALDOA was the only gene consistently and prominently upregulated at both mRNA and protein levels in LUAD cells and served as a core high-risk factor in our five-gene MDrisk signature, we selected ALDOA for further functional validation to explore its biological role in LUAD progression. Thus, ALDOA represented a rational and prioritized candidate for initial functional characterization. Stable knockdown of ALDOA was established in A549 and H358 cells and verified by Western blot (Figure 9G). Functional assays demonstrated that ALDOA knockdown significantly suppressed cell proliferation, as shown by CCK-8 and EdU incorporation assays (Figures 9H–K). Moreover, silencing ALDOA markedly inhibited cell migration and invasion, as validated by wound-healing and transwell assays, respectively (Figures 9L–O).

**FIGURE 10:**
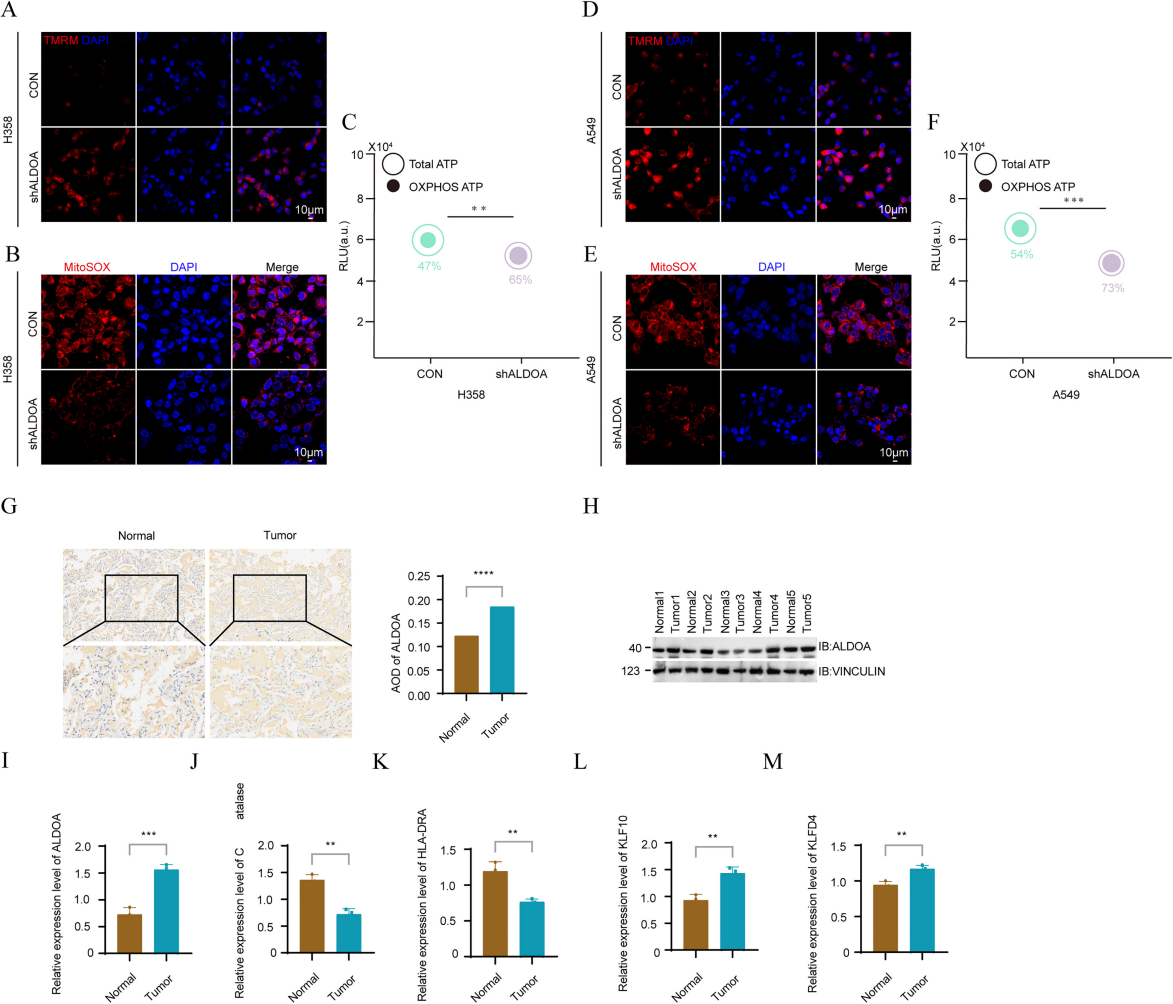
Mitochondrial consequences of ALDOA knockdown in lung adenocarcinoma (LUAD) cells and expression pattern of MDrsik genes in LUAD patient samples. **(A)** Representative TMRM confocal images of H358-shCon and H358-shALDOA cells showing increased ΔΨm upon ALDOA knockdown. **(B)** Representative MitoSOX Red confocal images of H358 cells showing decreased mitochondrial superoxide after ALDOA silencing. **(C)** OXPHOS-dependent ATP production measured by the Glycolysis/OXPHOS Assay Kit (Dojindo) in H358 cells. **(D–F)** Corresponding TMRM, MitoSOX, and ATP data for A549 cells. **(G)** IHC and quantification analysis of ALDOA in LUAD tissues and adjacent normal tissues. **(H)** Western blot analysis of ALDOA protein levels in LUAD patient tissues. **(I–M)** Box plots demonstrating the mRNA expression levels of the five MDrisk genes in LUAD patient samples. **p < 0.01; ***p < 0.001; ****p < 0.0001.

To establish a direct mechanistic link between ALDOA and mitochondrial energy metabolism, we further evaluated mitochondrial membrane potential (ΔΨm), mitochondrial reactive oxygen species (ROS) levels, and oxidative phosphorylation capacity following ALDOA silencing. Confocal imaging of TMRM staining revealed that ALDOA-knockdown A549 and H358 cells displayed noticeably brighter fluorescence compared with their respective control cells, indicating mitochondrial hyperpolarization (Figures 10A, D). In parallel, MitoSOX Red staining showed markedly weaker fluorescence intensity in shALDOA cells, suggesting reduced mitochondrial superoxide accumulation upon ALDOA depletion (Figures 10B, E). Furthermore, quantitative measurement of OXPHOS-dependent ATP production using the Glycolysis/OXPHOS Assay Kit demonstrated that ALDOA knockdown significantly decreased ATP levels in both A549 and H358 cells (Figures 10C, F). Collectively, these results indicate that ALDOA, although canonically regarded as a cytoplasmic glycolytic enzyme, exerts a profound influence on mitochondrial function, including ΔΨm maintenance, redox homeostasis, and oxidative phosphorylation capacity.

Clinical LUAD tumor specimens were collected to further validate the clinical implications of the signature gene expression patterns. Immunohistochemistry (IHC) and Western blot analyses demonstrated markedly upregulated ALDOA expression in LUAD tumor tissues compared with normal control samples (Figures 10G, H). Consistent with the *in vitro* experimental findings, the other four signature genes also displayed identical expression trends in clinical specimens (Figures 10I–M). These clinical sample-derived evidence strongly supports the critical roles of these genes in LUAD diagnosis and prognostic assessment. Integrated analyses of *in vitro* cellular phenotypes and human tissue validation collectively indicate that ALDOA functions as a key oncogenic regulator in LUAD. By facilitating the proliferative, migratory, and invasive capacities of LUAD cells, ALDOA drives tumor malignant progression. However, it should be noted that only ALDOA was subjected to complete *in vitro* functional characterization, and the biological roles of the remaining four genes were not individually or combinatorially validated in cellular experiments. Therefore, these findings should not be overgeneralized to the entire five-gene signature. These findings further validate the biological credibility and clinical translational value of the five-gene-based MDrisk signature.

## 4 Discussion

Lung adenocarcinoma remains a major cause of cancer-related mortality worldwide, marked by high intratumor heterogeneity, frequent therapeutic resistance, and unsatisfactory prognosis (17). Mitochondrial energy metabolism is increasingly recognized as a core regulator of tumor malignant progression and treatment response (18). However, the integrated link between mitochondrial metabolism, drug resistance, immune microenvironment, and clinical outcome in LUAD has not been fully elucidated. In this study, we constructed a novel mitochondrial metabolism-drug resistance prognostic signature, termed MDrisk, using single-cell transcriptomics, WGCNA, and multi-algorithm machine learning, and systematically validated its prognostic value, immune characteristics, genomic alterations, and biological function.

Using single-cell RNA sequencing and WGCNA, we identified gene modules closely associated with MRGs and DRGs, supporting the critical role of metabolic reprogramming in therapeutic failure. Through multi-step screening and LASSO-Cox regression, we established a five-gene signature (KLF4, KLF10, CAT, ALDOA, HLA-DRA) and selected the optimal model via 101 machine learning combinations. The MDrisk signature exhibited stable and powerful prognostic performance in training, test, and independent GEO cohorts, and was confirmed as an independent prognostic factor after adjusting for age, gender, and TNM stage. The integrated nomogram further improved the accuracy of individualized survival prediction, suggesting its potential for clinical application.

To contextualize the predictive performance of our model, we compared MDrisk with representative published LUAD multi-omics and machine learning prognostic signatures (Supplementary Table 2). The MDrisk model achieved a mean C-index of 0.653 across training, internal test, and external validation cohorts, which is within the range (0.60–0.75) reported by most published LUAD signatures based on transcriptomic data. Importantly, the unique contribution of MDrisk lies in its dual integration of mitochondrial energy metabolism and drug resistance gene modules—a biological perspective that has not been captured by previous signatures that primarily rely on traditional clinicopathological factors or single biological themes. This metabolic–resistance dual perspective, combined with rigorous multi-algorithm benchmarking, confers added value in both mechanistic interpretability and predictive robustness.

Although only ALDOA and CAT are intuitively linked to core metabolic functions, accumulating evidence supports the connection of all five genes to mitochondrial energy metabolism and drug resistance. ALDOA, encoding aldolase A, is a rate-limiting glycolytic enzyme that drives the Warburg effect and enhances aerobic glycolysis, lactate production, and ATP generation (19). Its overexpression has been mechanistically linked to EGFR/MAPK and PI3K/Akt pathway activation, cell cycle progression, and cytoskeletal remodeling in non-small cell lung cancer (20). Elevated ALDOA is significantly associated with advanced tumor stage, metastasis, and poor prognosis in LUAD (21). CAT encodes catalase, a key mitochondrial and peroxisomal antioxidant enzyme that detoxifies hydrogen peroxide (22). Dysregulation of CAT disrupts redox homeostasis, leading to elevated intracellular ROS levels that can trigger mitochondrial dysfunction and DNA damage, both of which are closely associated with chemoresistance in multiple cancers (23). KLF4 regulates mitochondrial oxidative phosphorylation and is involved in maintaining mitochondrial homeostasis; its dysregulation has been implicated in cisplatin resistance in lung cancer and stemness maintenance (24). KLF10 is a TGF-β-inducible transcription factor that modulates mitochondrial apoptosis pathways and cellular responses to oxidative stress; its altered expression contributes to resistance to platinum-based chemotherapies (25). HLA-DRA, encoding the alpha chain of MHC class II molecules, regulates immune recognition within the TME (26, 27). Mitochondrial dysfunction can impair antigen processing and presentation via reduced ATP supply and increased oxidative stress, thereby modulating immune checkpoint expression and contributing to immune evasion and resistance to immunotherapy (28, 29). Collectively, these five genes form a functional network linking mitochondrial energy metabolism to drug resistance through redox imbalance, metabolic reprogramming, apoptotic evasion, and immune suppression. It is important to note that the inclusion of CAT, KLF4, KLF10, and HLA-DRA in the final signature was primarily driven by machine-learning optimization and statistical performance rather than direct mechanistic evidence linking them to mitochondrial metabolism in LUAD. Their predictive value should be interpreted as a result of computational selection, and the mechanistic basis of these genes in mitochondrial dysfunction and drug resistance warrants further experimental investigation.

Immune and enrichment analyses revealed that high MDrisk scores were significantly correlated with immunosuppressive tumor microenvironment, including reduced immune cell infiltration, impaired antigen presentation, and upregulated immune checkpoint molecules. Meanwhile, high-risk patients exhibited higher TMB and distinct CNV patterns, which may contribute to heterogeneous therapeutic responses. Drug sensitivity analysis indicated that MDrisk could computationally predict responsiveness to multiple chemotherapeutic and targeted agents, providing a reference for personalized treatment selection.

In conclusion, our study established a robust MDrisk signature that effectively predicts prognosis, immune landscape, and computationally estimated therapeutic response in LUAD. These findings deepen the understanding of metabolic-immune interactions in LUAD and provide a promising tool for further research in precision medicine. Future prospective studies are needed to validate the clinical utility of MDrisk and explore targeted therapeutic strategies to overcome metabolism-mediated drug resistance.

Compared with previously published LUAD signatures, our study has several distinguishing features that may be of interest. Compared with previously published LUAD signatures, our study offers several distinguishing features. While most existing signatures focus on single biological themes such as immunogenic cell death, ferroptosis, or glycolytic pathways (30, 31), the MDrisk model uniquely integrates two functionally interconnected yet independently critical dimensions—mitochondrial energy metabolism and drug resistance—within a unified analytical framework. This dual-perspective design provides enhanced biological interpretability and captures the interplay between metabolic dysregulation and therapeutic failure. Methodologically, our 101-algorithm benchmarking pipeline represents a more comprehensive and unbiased approach compared to the single-algorithm or limited-algorithm frameworks adopted by most previous studies. Furthermore, our analysis uniquely spans single-cell transcriptomics and bulk transcriptomic levels, a feature absent in the vast majority of prior LUAD prognostic studies.

However, several limitations of this study should be acknowledged. First, all sequence data were retrospectively collected from public databases, and potential selection bias could not be fully avoided. Second, the MDrisk model was constructed and validated based on transcriptomic data, but its predictive value needs to be further verified in prospective multi-center clinical cohorts. Third, only the biological function of ALDOA was explored *in vitro*; the detailed molecular mechanisms underlying the five-gene signature, as well as the crosstalk between mitochondrial metabolism, drug resistance, and tumor immunity, remain to be further investigated *in vivo*. Specifically, the biological roles of KLF4, KLF10, CAT, and HLA-DRA were not individually or combinatorially validated in cellular or animal experiments, and the findings from ALDOA alone should not be extrapolated to the entire signature without caution. Fourth, our immunotherapy-related predictions—including TIDE scores, ESTIMATE analyses, immune cell deconvolution, and drug sensitivity estimates—are derived exclusively from computational algorithms and *in silico* inference. These findings, although suggestive, do not constitute direct clinical evidence of differential immunotherapy benefit and must be interpreted with appropriate caution. Direct validation in clinical cohorts of LUAD patients who have received defined immunotherapy or targeted therapy regimens is required before MDrisk can be recommended as a predictive biomarker for clinical treatment decisions. Fifth, the single-cell transcriptomic analysis is constrained by the gene panel coverage of the underlying platform, as targeted sequencing panels may omit a substantial proportion of the global transcriptome. Consequently, the identified candidate genes and subsequent cell-state characterizations should be regarded as the most reliable proxies identifiable within the available data rather than a comprehensive representation of all molecular determinants of mitochondrial metabolic dysfunction and drug resistance. Finally, functional validation of ALDOA was limited to two-dimensional *in vitro* cellular phenotypes (proliferation, migration, and invasion), which are insufficient to fully recapitulate the complex metabolic-immune crosstalk occurring within the genuine tumor microenvironment. *In vivo* functional studies using ALDOA-silenced LUAD cells in subcutaneous xenograft and metastatic mouse models are warranted to further elucidate the mechanistic role of ALDOA in mitochondrial metabolic rewiring and therapeutic resistance under physiologically relevant conditions.

## Data Availability

The original contributions presented in this study are included in the article/Supplementary material, further inquiries can be directed to the corresponding authors.
