# Supplementary material for: The role of mitochondrial energy metabolism in drug resistance and prognosis of lung adenocarcinoma: a multi-omics and machine learning strategy for predictive and personalized therapy
Source: Front Med (Lausanne). 2026 Jul 31;13:1892773. doi: 10.3389/fmed.2026.1892773 (PMC13472987; doi:10.3389/fmed.2026.1892773)
Supplement: Supplementary file 1 [file Table_2.docx]

Supplementary Material

# 1 Supplementary Table The primer sequences used in this study.

| **Gene Symbol** | **Primer Sequences** |
| --- | --- |
| *ALDOA* | *ALDOA* (F): CCCAAGTTATCAAATCCAAG  *ALDOA* (R): GGTCACTCAGAGCCTTGTAG |
| *CAT* | *Catalase* (F): AATCAGAAGGCAGTCCTCCC  *Catalase* (R): TCGGGGAGCACAGAGTGTAC |
| *HLA-DRA* | *HLA-DRA* (F): AAGAAGGAGACGGTCTGG  *HLA-DRA* (R): TGGTCCCAATAATGATGC |
| *KLF10* | *KLF10* (F): CAGCCAACCATGCTCAACTTCG  *KLF10* (R): TTGAGGATGCTGGCTGCTTTCA |
| *KLF4* | *KLF4* (F): CAGTCGCAAGTCCCCTCTCT  *KLF4* (R): TATCAAGAGCTCATGCCAC |
| *GAPDH* | *GAPDH* (F): GCACCGTCAAGGCTGAGAAC  *GAPDH* (R): TGGTGAAGACGCCAGTGGA |
